# Cross-Cultural Adaptation and Validation of the Portuguese Version of the SARC-F in Community-Dwelling Older Adults

**DOI:** 10.3390/diagnostics14111096

**Published:** 2024-05-24

**Authors:** Margarida Isabel Boteta-Gomes, Agustín Aibar-Almazán, Fidel Hita-Contreras, Nuno Eduardo Marques de Loureiro, Vânia Azevedo Ferreira Brandão-Loureiro

**Affiliations:** 1 Department of Arts, Humanities and Sport, Polytechnic Institute of Beja, 7800-295 Beja, Portugalnloureiro@ipbeja.pt (N.E.M.d.L.);; 2 SPRINT—Sport Physical activity and health Research & INnovation CenTter, 7800-295 Beja, Portugal; 3 Department of Health Sciences, Faculty of Health Sciences, University of Jaén, 23071 Jaén, Spain; 4 ISAMB, University of Lisbon, 1649-028 Lisbon, Portugal

**Keywords:** SARC-F, Portuguese, sarcopenia, psychometric, validation

## Abstract

(1) Background: The goal of this study was to analyze the reliability and validity of the Portuguese version of the SARC-F in older adults. (2) Methods: A total of 100 participants (77.1 ± 7.36 years, 73% women) were included in the study. In a first phase, the Portuguese SARC-F was adapted following the standardized forward–backward translation procedure, and internal consistency as well as inter-rater and test–retest reliability of the Portuguese SARC-F were analyzed. Secondly, clinical validation was evaluated by comparing the SARC-F total score with five operational definitions of sarcopenia and with other sarcopenia-related measurements. Discriminant validity, with respect to low muscle mass and strength and physical function were analyzed. (3) Results: The Portuguese SAR-F showed acceptable internal consistency (Cronbach α = 0.82), excellent inter-rater reliability (total score), and substantial to excellent test–retest reliability (ICC = 0.891 for the total score). Specificity ranged from 72.5% (FNIH) to 73.4 (IGWS), and negative predictive values went from 91.8% (EWGSOP1) to 97.3% (FNIH), but low sensitivity and positive predictive value were observed. The Portuguese SARC-F showed a moderate ability to discriminate people with low muscle strength (AUC = 0.78) and gait speed (AUC = 0.89). (4) Conclusions: The Portuguese SARC-F is a valid and reliable tool for ruling out sarcopenia in community-dwelling older adults and can discriminate between people with low handgrip strength and gait speed.

## 1. Introduction

The aging of the world population is a proven fact, and in fact, it is estimated that, between 2015 and 2050, the proportion of people over 60 years will rise from 12% to 22% [1]. The term sarcopenia was initially used to describe the loss in muscle mass with advancing age [2]. Since then, new diagnostic criteria have been added to the definition of sarcopenia, such as muscle strength and physical performance, and it was recognized as a disease by the International Classification of Diseases, Tenth Revision, Clinical Modification (code M62.84) [3].

Currently, there are several definitions of sarcopenia according to the different study groups that consider different criteria and cutoff values. Some of the most commonly used diagnostic criteria are those proposed by the European Working Group on Sarcopenia in Older People, in 2010 (EWGSOP1) [4], and updated in 2018 (EWGSOP2) [5]. In 2014, the Asian Working Group on Sarcopenia (AWGS) [6] proposed a diagnostic algorithm based on Asian data which was updated in 2019 (AWGS-2019) [7]. There are also criteria proposed for sarcopenia diagnostic by the International Working Group on Sarcopenia (IWGS) [8], or the Foundation for the National Institutes of Health (FNIH) Sarcopenia Project [9]. It has been estimated that the prevalence of sarcopenia worldwide ranges from 10–16% of the older adults [10], but it may vary depending on the definition criteria or the studies analyzed [11].

Sarcopenia has been related to numerous adverse outcomes, including poor health-related quality of life, cognitive decline or hypertension and cardio-cerebrovascular disease or even increased mortality [12,13,14,15,16,17]. Moreover, sarcopenia has been associated with higher intensive care unit admission and increased hospital stays, severity of disease and risk of mortality among COVID-19 patients [18].

The diagnosis of sarcopenia in daily clinical practice is not easy, since, in many cases, the time and specific equipment required is not available [19]. Therefore, it is necessary to develop simple, easy-to-use, validated tools that allow for rapid screening for sarcopenia.

The SARC-F (strength, assistance in walking, rise from a chair, climb stairs, and falls) questionnaire is valid and internally consistent for screening persons at risk for adverse outcomes from sarcopenia [20,21], and it stands as one of the best tools to evaluate sarcopenia in every day practice [22]. The EWGSOP2 recommends the SARC-F as a way to introduce the assessment and treatment of sarcopenia into clinical practice, and it can be easily administered in community healthcare and other clinical settings [5].

The SARC-F was originally developed in English, and to the date, it has been translated and validated for many languages and populations [23,24,25,26]. A translation of the Portuguese versions of the SARC-F, together with the FRAIL Scale, were performed in 2021 [27], but to the best of our knowledge, the analysis of the reliability and clinical validity of the cross-culturally adapted Portuguese version of the SARC-F has not been carried out. This questionnaire has been validated for the Brazilian Portuguese spoken population [28], but despite the fact that Portugal and Brazil share a common language, several cultural and dialectal differences must be taken into consideration.

In view of the above, the objective of this study was to perform the translation and cross-cultural adaptation of the Portuguese version of the SAC-F and evaluate its clinical validity in Portuguese community-dwelling adults aged 65 years or older.

## 2. Materials and Methods

### 2.1. Study Design and Participants

A total of 100 older adults took part in this analytical cross-sectional study that was carried out from January 2024 to February 2024. The sample size was considered appropriate according to the recommendations described by the Sarcopenia Special Interest Group of the European Geriatric Medicine Society (EuGMS) for the SARC-F validation studies (at least 50–100 community-living men and women aged 65 years or older in every country) [22] and was similar to other SARC-F validations [26,29]. Recruitment was conducted by contacting people from different associations from different cities of Portugal such as Beja (Laboratório de Actividade Física e Saúde, IPBEJA-Projeto UP AGAIN SENIOR and Universidad Sénior de Beja), Serpa (Programa Gente em Movimento), Cuba (Universidad Sénior de Cuba) and Baleizão (Centro Social Nossa Senhora da Graça). This study was approved by the ethics committee of Polytechnic institute of Beja (CEIPBeja № 03/2019). This study was performed following the Declaration of Helsinki, good clinical practices, and all applicable laws and regulations, and a written informed consent to participate was provided by all the participants.

Inclusion criteria were being 65 years or older, native Portuguese speakers, able to understand the purpose of this study and complete the questionnaires and walk independently or with aids in safety conditions. Participants were excluded if they were bed-bound, had chronic and/or severe medical condition that could influence their answers to the questionnaire, had health contraindications for bioelectrical impedance (such as metallic implants or pacemakers), or did not give their willingness to take part in the present study.

### 2.2. Procedure

The translation and validation process has been carried out following the guidelines described by the Sarcopenia Special Interest Group of the EuGMS, which consists of two consecutive phases [22]. For this study, permission was obtained from Professor Malmstrom. In the first phase, the translation and cross-cultural adaptation procedure into Portuguese and the reliability evaluation (inter-rater and test–retest) were performed. The consensus preliminary Portuguese version of the SARC-F was obtained from the original version by 2 bilingual experts working together with clinical professionals who were familiar with this topic. Given that metric units are employed in Portugal, “10 pounds” was replaced by “about 5 kg” in the item 1 of the SARC-F. The Portuguese version was applied to 10 participants (5 men and 5 women) to analyze doubts and suggestions regarding the questionnaire. After this, the Portuguese version of the SARC-F was translated back into English and was compared with the original version to determine semantic and linguistic equivalence. Inter-rater reliability was evaluated by 2 independent experts in 20 participants (10 men and 10 women), and in order to assess test–retest reliability, the questionnaire was completed again by another 20 participants (not duplicated) two weeks later.

In the second phase, the clinical validation of the Portuguese version of the SARC-F questionnaire was assessed by evaluating the ability to discriminate between people with and without sarcopenia according to the operational definitions of sarcopenia described by the EWGSOP1 [4], EWGSOP2 [5], IWIWGS-2019 [7], AWGS [8], and FNIH [9] (Table 1). The associations between the total score of Portuguese of the SARC-F and other body composition and sarcopenia-related parameters were also determined.

### 2.3. Outcome Measures

Sociodemographic data, including sex, age and education level, were collected. A bioelectrical impedance analyzer (Tanita^®^ BC-601, Tokyo, Japan) and a stadiometer (SECA^®^ 213, Seca, Ltd., Hamburg, Germany) were used to determine weight and height, respectively; body mass index (BMI) was obtained by dividing the participant’s weight (kg) by the participant’s height (m^2^). A BMI < 25 kg/m^2^ indicates normal weight, between 25 and 29.9 kg/m^2^ overweight, and BMI ≥ 30 kg/m^2^ obesity [30]. Calf diameter was measured 10 cm below the tibial tuberosity with an inextensible tape (SECA^®^ 201, Seca, Ltd., Hamburg, Germany).

The SARC-F questionnaire [20] is an inexpensive and convenient method for sarcopenia risk screening [5]. It consists of five domains or items: strength, walking across a room, rising from a chair, climbing stairs, and number of falls in the last year. Each domain is scored from 0 to 2, and the questionnaire provides a total score that ranges from 0 to 10, with greater scores indicating a higher risk of sarcopenia. A cut-off score of ≥4 indicates risk of sarcopenia [21].

Handgrip strength was assessed by a Jamar^®^ J00105 hydraulic hand dynamometer (Jamar, Irvington, NY, USA). Participants were standing with their arms parallel to their trunk with the dynamometer facing outwards the body and were instructed to exert maximum grip three times on each hand with a rest period of one minute between trials. Handgrip strength was evaluated three times on each hand alternately, and the highest value was noted regardless of the dominant hand [31]. As for muscle mass, appendicular skeletal muscle mass (ASM) was calculated by bioelectrical impedance (Tanita^®^ BC-601, Tokyo, Japan). Participants stood on the two metallic electrodes on the measuring platform barefoot with their socks or stockings removed and the soles of their feet clean, without eating or exercising for about three hours [32]. ASM index (ASMI) was determined by dividing ASMI by the participant’s height in square meters (kg/m^2^) and by BMI. Usual gait speed was evaluated by the 6 m gait speed test [33]. Participants were required to walk ten meters at a maximum speed (without running). The time was determined from the third to the eighth meter. The first and the last two meters, which refer to the acceleration and deceleration periods, respectively, were not included. Speed was obtained by dividing six meters by the time to walk this distance(s). The timed up and go (TUG) test was also used to assess physical performance, where participants were required to rise from a chair, walk three meters, turn around, walk back, and sit down again [34].

### 2.4. Statistical Analysis

Statistical analysis and data management were performed in the SPSS 20.0 statistical package (SPSS Inc., Chicago, IL, USA). The level of statistical significance was set at *p* < 0.05. The Kolmogorov–Smirnov test was used to determine normality. Frequencies and percentages, as well as mean and standard deviation (SD), were employed for the categorical and continuous variables, respectively. According to the variables, Student t or chi-square tests and Spearman’s correlation analysis were performed. Cronbach’s α coefficient was employed to evaluate the internal consistency of the questionnaire, where values ≥ 0.7 were considered acceptable for general research purposes [35]. The Spearman’s test was used to evaluate the item total score correlations. Spearman’s correlation coefficients (rho) ≥ 0.81 were considered as excellent, between 0.61 and 0.8 as very good, between 0.41 and 0.6 as good, between 0.21 and 0.4 as acceptable, and ≤0.2 as insufficient [36]. Inter-rater and test–retest reliability were determined by the intraclass correlation coefficient (ICC2,1) by Shrout and Fleiss. Values < 0.4 were considered poor, moderate between 0.4 and 0.75, substantial between 0.75 and 0.9, and >0.9 were classified as excellent [37]. As for the clinical validation of the Portuguese version of the SARC-F, sensitivity, specificity, positive predictive value (PPV), negative predictive value (NPV) and accuracy of the Portuguese SARC-F groups (cut-off ≥ 4) compared to the presence of sarcopenia according to the EWGSOP1, EWGSOP2, IWGS-2019, AWGS, and FNIH were obtained. The Spearman‘s correlation analysis was used to analyze the validation of the SARC-F total score against other measurements related to sarcopenia, and a multivariate linear regression model (stepwise method) was used to determine the independent associations. To determine the effect size, the coefficient of multiple determination (adjusted-R^2^) was used. Values were considered as insignificant when <0.02, small when 0.02–0.15, medium when 0.15–0.35, and large when >0.35 [38]. A receiver operating characteristic (ROC) curve analysis was used to determine the accuracy of the Portuguese SARC-F total score in discriminating between participants with and without low handgrip strength, ASMI, and gait speed. Values of the area under the ROC curve (AUC) > 0.9 were considered as high, between 0.7 and 0.9 as moderate, and between 0.5 and 0.7 as low [39].

## 3. Results

**Figure 1:**
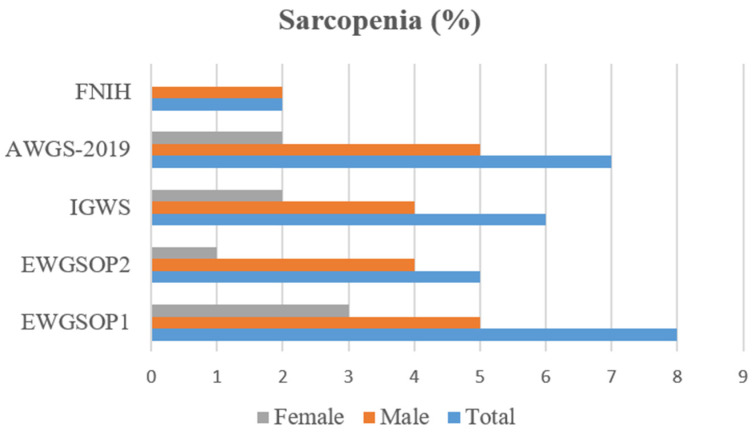
Percentage of sarcopenia according to the different operational definitions. AWGS: Asian Working Group for Sarcopenia. EWGSOP1: European Working Group on Sarcopenia in Older People. EWGSOP2: European Working Group on Sarcopenia in Older People—revised. FNIH: Foundation for the National Institutes of Health. IWGS: International Working Group on Sarcopenia.

The descriptive characteristics of the 100 participants (73% women) are displayed in Table 2. The mean age was 77.07 ± 7.36 years, most of the participants had a basic education level (77%) and were married (66%). As for the body composition and sarcopenia-related parameters, women had a significantly higher fat-mass percentage (*p* < 0.001), while men had significantly greater values of handgrip strength, ALMI-height^2^, and ALMI-BMI (all *p* < 0.001). According to the SARC-F total score, 27% of the participants were at risk of sarcopenia (SARC-F ≥ 4), and women a had significantly greater SARC-F total score (*p* < 0.001). When analyzing the prevalence of sarcopenia (Figure 1), the highest and lowest percentages were seen in the diagnosis according to EWGSOP1 (8%) and FNIH (2%), respectively. In our sample, men had a higher rate of sarcopenia regardless of the diagnostic criteria.

### 3.1. Reliability

As for the internal consistency, the analysis showed a Cronbach α value of 0.82 for the total score of the Portuguese SARC-F, which determines an acceptable level of internal consistency (Cronbach α ≥ 0.7). Item-to-total score correlation was also performed (Table 3) and showed significant positive correlations (all *p* < 0.001), with Spearman’s coefficient values ranging from 0.63 (item 5) to 0.87 (item 4).

Regarding reliability, the inter-rater reliability analysis was carried out on 20 different participants (76 ± 6.32 years, 50% women), and we obtained an ICC value of 0.96 for the SARC-F total score, which indicate an excellent inter-rater reliability. The test–retest reliability analysis revealed substantial ICC values for item 3 (ICC = 0.81), item 1 (ICC = 0.83), item 5 (ICC = 0.85), and the total score (ICC = 0.89), while excellent correlations were observed for items 4 (ICC = 0.93) and 2 (ICC = 1).

### 3.2. Clinical Validity

The specificity of the Portuguese SARC-F according to the different diagnostic criteria of sarcopenia were similar (Table 4), ranging from 72.5% (FNIH) to 73.4 (IGWS), while negative predictive values went from 91.78% (EWGSOP1) to 97.3% (FNIH). On the other hand, low scores were observed with respect to sensitivity, with percentages ranging from 0 to 33.3% (for FNIH and IGWS, respectively) and positive predictive values, ranging from 0% (FNIH) to 7.41% (EWGSOP1, IGWS, and AWGS-2019). Accuracy levels were similar (69–71%), and the values regarding the area under the ROC curve went from 0.52 (FNIH) to 0.634 (IGWS).

**Figure 2:**
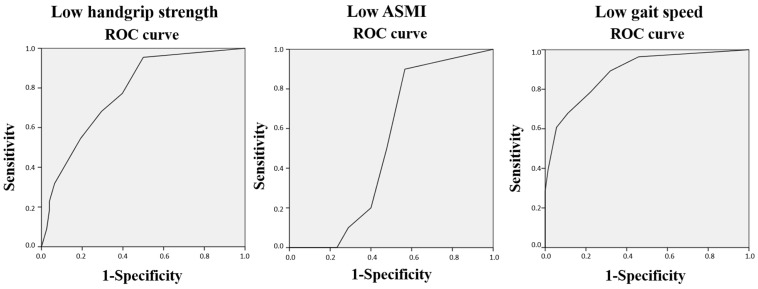
The ROC curve of the SARC-F total score for discriminating between participants with and without low muscle strength, mass, and gait speed. ASMI: Appendicular Skeletal Muscle Mass Index. ROC: Receiver Operating Characteristic.

Concerning the associations between the total score of Portuguese of the SARC-F and sarcopenia-related parameters (Table 5), our findings revealed significant (all *p* < 0.001) and good correlations with handgrip strength, gait speed, and the TUG test (Rho Spearman’s Correlation Coefficients of −0.40, −0.68, and 0.62, respectively). As for muscle mass, no significant correlations were observed with ALMI. However, calf diameter was correlated to the SARC-F total score (rho = 0.26, *p* = 0.01). When the multivariate linear regression analysis was performed (Table 6), muscle strength (which indicates probable sarcopenia according EWGSOP2) and gait speed (severity of sarcopenia) showed independent associations, with an adjusted-R^2^ of 0.50 for the model, which indicated a large effect size. In the analysis of the discriminative ability of the Portuguese SARC-F total score with respect to sarcopenia-related parameters according to the EWGSOP2 (Figure 2), our results showed that the AUC for low handgrip strength (22% of the sample, 63.4% women) and low gait speed (28% of the participants, 75% women) were 0.78 (95% confidence interval = 0.68–0.88) and 0.89 (95% confidence interval = 0.81–0.96), respectively, while for low ASMI (10% of the participants, 40% women), it was 0.52 (95% confidence interval = 0.40–0.64).

## 4. Discussion

The objective of this study was to perform the cross-cultural adaptation and clinical validation of the Portuguese version of the SARC-F for a population of community-dwelling Portuguese older adults. Our findings show that the Portuguese SARC-F showed adequate reliability and clinical validity to be employed for the screening of sarcopenia in Portuguese older adults. It is also a valid instrument to identify people with low handgrip strength and gait speed.

The SARC-F total score of the present study was 2.25 ± 2.61, with 27% of the participants at risk of sarcopenia (SARC-F ≥ 4), values that are between those described in previous validation articles such as those of Tsekoura et al. [25] in Greek people (2.8 ± 1.9 and 32.3%), or Parra-Rodríguez et al. [19] in Mexican people (1.95 ± 1.9 and 19.5%). On the other hand, 4.58% and 3.38% of the participants with a SARC-F total score ≥ 4 were described in the Thai [40] and Japanese [23] validations, respectively, where lower values could be explained by cultural and lifestyle factors. Our analysis also showed that women had a significantly higher SARC-F total score, which is in line with the findings described by Krzymińska-Siemaszko et al. [41] and Perna et al. [26] in the in the Polish and Italian validations, respectively.

The lowest prevalence of sarcopenia was observed according to the FNIH criteria (2%), which was expected given that the three diagnostic criteria are required (low values of muscle mass, strength, and physical performance). The other prevalence values were ranging from 5% (EWGSOP2) to 8% (EWGSOP1). As mentioned, low muscle mass, muscle strength, and physical fitness are the three parameters usually evaluated for sarcopenia diagnosis proposed by the different international working groups, but there are several variations regarding the diagnostic tools, cut-off points, or diagnostic criteria. For instance, Liu et al. [42] found that, in Chinese adults ≥ 50 years, the prevalence of sarcopenia according to different operational definitions were 11.8% (EWGSOP2), 18.1% (FNIH), 22.8% (AWGS 2019), 24.1% (IWGS), and 57.1% (EWGSOP1). However, there are other factors such as age group, gender, geographical areas, ethnicities, or locations that may affect the prevalence of sarcopenia [43,44]. A meta-analysis published by Petermann-Rocha et al. [45] described that the prevalence of sarcopenia varied from 10% to 27% in adults aged 60 years and over, where the highest prevalence was found in Oceania (EWGSOP1) and the lowest in Europe (EWGSOP2). Women had a higher prevalence according the IWGS (17% vs. 12%), and men when employing the EWGSOP2 (11% vs. 2%). Our study found that men showed a higher prevalence of sarcopenia under all the operational diagnostic criteria, and the greater difference was observed when EWGSOP2 (4% vs. 4%) and AWGS-2019 (5% vs. 2%) were followed.

The Portuguese SARC-F showed acceptable internal consistency (Cronbach α value = 0.82), which is in line with those described by other validations such as the Spanish [29], the Romanian [46], or the Polish versions [41] (Cronbach α values of 0.78, 0.76, and 0.7, respectively), while on the other hand, lower internal consistency was obtained in the Italian [26] or the Japanese [23] versions of the SARC-F (Cronbach α values of 0.67 and 0.61, respectively). Previous SARC-F validations have found significant item-to-total correlations, with the items 2 (assistance with walking) and 1 (strength) showing the lowest and the highest correlations, respectively [19,41]. In the present study, the analysis showed that all the questions of the SARC-F significantly correlated with the total score, where a higher correlation was observed for the question 4 (climbing stairs) and lower correlations for questions 2 (assistance with walking) and 5 (falls).

In order to assess test–retest reliability, the Portuguese SARC-F was administered again to a subsample of 20 participants after two weeks. This time interval was recommended by the EuGMS [22] and used in previous SARC-F validations. Our analysis revealed substantial to excellent ICC values for the SARC-F questions, with an ICC of 0.89 for the SARC-F total score. These findings are similar but slightly lower than the values described by Krzymińska-Siemaszko et al. [41] in the Polish validation (ICC = 0.923), but higher than those indicated by Parra-Rodríguez et al. [19] in the Spanish validation for Mexican population, and by Beaudart et al. [47] in the French version (ICC values of 0.80 and 0.86, respectively). Our findings also revealed an excellent inter-rater reliability for the Portuguese SARC-F total score, which is in agreement with other validation studies [25,41].

With regard to the clinical validation of the Portuguese SARC-F, our results showed lower values of sensitivity but greater specificity in the diagnosis of sarcopenia under the five diagnosis criteria. Additionally, weak positive predictive values were found, as well as high negative predictive values. These results are in agreement with the findings described in previous validation studies [19,25,41,47] and confirm the Portuguese SARC-F as a valid and capable tool for determining the absence of sarcopenia. The Portuguese SARC-F scores could be considered as low, especially in men, where all total scores were <4, and regarding the items, the highest score observed was two and only on one occasion (item 4, climb stairs). This could be due to the high level of physical functioning of the participants, which may also influence the low prevalence of sarcopenia and may have an effect on our results.

As for the comparison between the SARC-F total score and other parameters related to sarcopenia and body composition, significant correlations have been described [24]. Our results are in line with those described by Kera et al. [23] and by Krzymińska-Siemaszko et al. [41] in the Japanese and Polish study validations, respectively, and showed significant correlations with all the studied variables, except for the ASMI values. When analyzing the discriminant ability of the SARC-F with low muscle mass (according the EWGSOP2 criteria), the AUC was low (0.52), which could be explained by the low percentage of participants with low ASMI (10%). But on the other hand, our results showed that the Portuguese SARC-F was able to distinguish between people with low handgrip strength (probable sarcopenia) and low gait speed (which indicates severity) with AUC of 0.78 and 0.89, respectively, which indicate moderate accuracy level. These results could be influenced by the proportion of men and women in this study, given that significant gender-related differences regarding handgrip strength, muscle mass, and fat-mass percentage were observed.

Some limitations of the present study should be considered. Bioelectrical impedance analysis, although it is supported by study groups such as the EWGSOP 1 and 2 or the AGWS, is not the most accurate method to determine muscle mass as compared to magnetic resonance imaging, computed tomography, or dual-energy X-ray absorptiometry, but it is recommended in the usual clinical care for its affordability and portability [5]. On the other hand, since the study was conducted on older adults from a specific geographical location, with only 27% of men, and thus, any generalization of these findings should be limited to people with similar characteristics, and future studies should be carried out on a general sample with a more balanced ratio between men and women from different geographical regions.

## 5. Conclusions

The SARC-F questionnaire was cross-culturally adapted and validated in community-dwelling Portuguese adults aged 60 years and older. Our results showed acceptable internal consistency, excellent inter-rater reliability, and substantial to excellent test–retest reliability. The specificity and negative predictive values make SARC-F a suitable instrument to identify sarcopenia in community-dwelling older adults according to different operational definitions. The SARC-F also showed good associations with body composition and sarcopenia-related parameters and was found to be a valid instrument for discriminating between people with low handgrip strength and gait speed, which indicate probable severe sarcopenia, respectively.

## Figures and Tables

**Table 1:** Diagnostic criteria of sarcopenia according to different operational definitions of sarcopenia.

	I. Low Muscle Strength (HGS) ^(1)^		II. Low Muscle Quantity (ASMI ^(2)^/h^2^, Except for FNIH ^(3)^, ASMI/BMI ^(4)^)		III. Low Physical Performance (Gait Speed)		Sarcopenia Diagnosis
	Men	Women	Men	Women	Men	Women	
EWGSOP ^(5)^	<30 kg	<20 kg	<7.4 kg/m^2^	<5.6 kg/m^2^	≤0.8 m/s		II + I or II + III
EWGSOP2 ^(6)^	<27 kg	<16 kg	<7 kg/m^2^	<5.5 kg/m^2^	≤0.8 m/s		I + II
IWGS ^(7)^	-	-	<7.23 kg/m^2^	<5.67 kg/m^2^	≤1 m/s		II + III
AWGS-2019	<27 kg	<18 kg	<7 kg/m^2^	<5.7 kg/m^2^	≤1 m/s		II + I or II + III
FNIH	<26 kg	<16 kg	<0.79	<0.51	≤0.8 m/s		I + II + III

^(1)^ HGS: Handgrip Strength. ^(2)^ ASMI: Appendicular Skeletal Muscle Mass Index. ^(3)^ FNIH: Foundation for the National Institutes of Health. ^(4)^ BMI: Body Mass Index. ^(5)^ EWGSOP1: European Working Group on Sarcopenia in Older People AWGS: Asian Working Group for Sarcopenia. ^(6)^ EWGSOP2: European Working Group on Sarcopenia in Older People—revised. ^(7)^ IWGS: International Working Group on Sarcopenia.

**Table 2:** Descriptive characteristics of the participants.

		All the Participants (*n* = 100)		Men (*n* = 27)		Women (*n* = 73)		*p*-Value
Age (years) ^a^		77.1	7.36	78.3	6.38	76.6	7.69	0.314
Marital status ^b^	Married	66	66	22	81.48	44	60.3	0.204
	Divorced/separated	6	6	1	3.7	5	6.85	
	widowed	23	23	4	14.8	19	26.0	
	Single	5	5	0	0	5	6.85	
Education ^b^	No level	8	8	2	7.41	6	8.22	0.497
	Basic	77	77	19	70.4	58	79.5	
	Secondary	8	8	4	14.8	4	5.48	
	Higher	7	7	2	7.41	5	6.85	
BMI ^(1)^ (kg/m^2^) ^a^		27.0	4.26	26.3	4.45	27.3	4.2	0.311
Fat mass percentage ^a^		32.8	9.7	26.7	9.17	35.1	8.94	<0.001
SARC-F ^(2)^ total score ^a^		2.25	2.61	1.07	1.17	2.68	2.86	<0.001
Calf circumference (cm) ^a^		35.3	3.34	35.4	3.53	35.3	3.29	0.944
ASMI ^(3)^-height^2^ (kg/m^2^) ^a^		7.32	1.06	7.92	1.13	7.1	0.94	<0.001
ASMI-BMI ^a^		0.67	0.13	0.83	0.1	0.61	0.08	<0.001
Handgrip strength (kg) ^a^		22.1	9.24	32.2	10.1	18.4	5.15	<0.001
TUG ^(4)^ test (s) ^a^		11.0	8.86	11.0	9.11	11.0	8.83	0.974
Gait speed (m/s) ^a^		1.19	0.51	1.27	0.59	1.16	0.48	0.326

^a^ Data presented as mean and standard deviation. ^b^ Data presented as frequency and percentage. ^(1)^ BMI: body mass index. ^(2)^ SARC-F: strength, assistance in walking, rise from a chair, climb stairs, and falls. ^(3)^ ASMI: appendicular skeletal muscle mass index. ^(4)^ TUG: timed up and go.

**Table 3:** Item-to-total score correlation of the Portuguese version of the SARC-F.

SARC-F Items	SARC-F ^(1)^ Total Score	
	Spearman’s Coefficient	*p*-Value
1. Strength	0.77	<0.001
2. Assistance with walking	0.67	<0.001
3. Rising from a chair	0.73	<0.001
4. Climbing stairs	0.87	<0.001
5. Falls	0.63	<0.001

^(1)^ SARC-F: strength, assistance in walking, rise from a chair, climb stairs, and falls.

**Table 4:** Diagnostic values of the Portuguese SARC-F.

	SARC-F ^(1)^					
	Se ^(2)^	Sp ^(3)^	PPV ^(4)^	NPV ^(5)^	Acc ^(6)^	AUC ^(7)^
Sarcopenia EWGSOP1 ^(8)^	25	72.8	7.41	91.9	69	0.56
Sarcopenia EWGSOP2 ^(9)^	20	72.6	3.7	94.5	70	0.58
Sarcopenia IGWS ^(10)^	33.3	73.4	7.41	94.5	71	0.64
Sarcopenia AWGS ^(11)^-2019	28.6	73.1	7.41	93.1	70	0.61
Sarcopenia FNIH ^(12)^	0	72.5	0	97.3	71	0.52

Values expressed as percentages. ^(1)^ SARC-F: strength, assistance in walking, rise from a chair, climb stairs, and falls. ^(2)^ Se: sensitivity. ^(3)^ SP: specificity. ^(4)^ PPV: positive predictive value. ^(5)^ NVP: negative predictive value. ^(6)^ Acc: accuracy. ^(7)^ AUC: area under the ROC curve. ^(8)^ EWGSOP1: European Working Group on Sarcopenia in Older People. ^(9)^ EWGSOP2: European Working Group on Sarcopenia in Older People—revised. ^(10)^ IWGS: the International Working Group on Sarcopenia. ^(11)^ AWGS: Asian Working Group on Sarcopenia. ^(12)^ FNIH: Foundation for the National Institutes of Health.

**Table 5:** Correlations of the Portuguese version of the SARC-F with body composition and sarcopenia-related parameters.

	SARC-F ^(1)^ Total Score	
	Spearman’s Coefficient	*p*-Value
BMI ^(2)^	−0.23	0.02
Fat mass percentage	−0.23	0.025
Calf circumference	−0.26	0.01
ASMI ^(3)^-height^2^	−0.12	0.248
ASMI-BMI	−0.11	0.264
Handgrip strength	−0.4	<0.001
TUG ^(4)^ test	0.62	<0.001
Gait speed	−0.68	<0.001

^(1)^ SARC-F: strength, assistance in walking, rise from a chair, climb stairs, and falls. ^(2)^ BMI: body mass index. ^(3)^ ASMI: appendicular skeletal muscle mass index. ^(4)^ TUG: timed up and go.

**Table 6:** Multivariate linear regression analyses.

		β_a_^(1)^	Β_b_^(2)^ (95% CI ^(3)^)	*p*-Value
SARC-F ^(4)^	Handgrip strength	−3.13	−0.61 (−3.92, −2.34)	>0.001
	Gait speed	−0.05	−0.19 (−0.10, −0.01)	0.018

^(1)^ β_a_: unstandardized coefficient. ^(2)^ Β_b_: standardized coefficient. ^(3)^ CI: confidence interval. ^(4)^ SARC-F: strength, assistance in walking, rise from a chair, climb stairs, and falls.

## Data Availability

The data shown in this study are available upon request from the corresponding author. The data are not available to the public, since taking into account the sensitive nature of all the questions asked in this study, all participants were guaranteed that the data obtained would be confidential and would not be shared.
